# *Momordica charantia* L.—Diabetes-Related Bioactivities, Quality Control, and Safety Considerations

**DOI:** 10.3389/fphar.2022.904643

**Published:** 2022-05-17

**Authors:** Serhat S. Çiçek

**Affiliations:** Department of Pharmaceutical Biology, Institute of Pharmacy, Kiel University, Kiel, Germany

**Keywords:** diabetes mellitus, anti-diabetic activity, triterpene saponins, momordicosides, cucurbitanoids, GLUT4, momordica charantia, bitter melon

## Abstract

*Momordica charantia* L. (Cucurbitaceae), commonly known as bitter gourd or bitter melon, is widely cultivated in many tropical and subtropical regions of the world, where its unripe fruits are eaten as a vegetable. Apart from its culinary use, *M. charantia* has a long history in traditional medicine, serving as stomachic, laxative or anthelmintic, and, most notably, for the treatment of diabetes and its complications. Its antidiabetic properties and its beneficial effects on blood glucose and lipid concentrations have been reported in numerous *in vitro* and *in vivo* studies, but the compounds responsible for the observed effects have not yet been adequately described. Early reports were made for charantin, a mixture of two sterol glucosides, and the polypeptide p-insulin, but their low concentrations in the fruits or their limited bioavailability cannot explain the observed therapeutic effects. Still, for many decades the search for more reasonable active principles was omitted. However, in the last years, research more and more focused on the particular cucurbitane-type triterpenoids abundant in the fruits and other parts of the plant. This mini review deals with compounds isolated from the bitter gourd and discusses their bioactivities in conjunction with eventual antidiabetic or adverse effects. Furthermore, methods for the quality control of bitter gourd fruits and preparations will be evaluated for their meaningfulness and their potential use in the standardization of commercial preparations.

## Introduction

Diabetes is one of the major health challenges of the 21st century. Currently an estimated 537 million adults suffer from diabetes, a number that is expected to rise by another 100 million until the end of this decade (International Diabetes Federation, 2021). With three out of four adults with diabetes living in low- or middle-income countries, their access to conventional antidiabetic drugs is restricted (International Diabetes Federation, 2021; Liu et al., 2021). Therefore, alternative remedies for the treatment of diabetes are of great importance.

One such remedy is *Momordica charantia* L. (Cucurbitaceae), an herbaceous vine cultivated throughout the tropics, where it is referred to as balsam pear, cundeamor (South America), karela (India), or goo-fah (Jamaica) (Walters and Decker-Walters, 1988; Raman and Lau, 1996). However, the two most common names are bitter gourd and bitter melon, indicating the consumption of its (unripe) fruits as a vegetable (Tan et al., 2016). Moreover, *M. charantia* has been used since ancient times in Traditional Chinese Medicine for treating high blood sugar and early signs of diabetes (Yin et al., 2008). The most popular ethnomedicinal preparations of the bitter gourd are karela juice, which is obtained by crushing and straining the unripe fruits, and cerasee, a decoction of the aerial parts of the plant (Bailey et al., 1986). Nowadays, capsules and tablets containing powdered drug or extracts are marketed as dietary supplements and can be purchased over-the-counter and from internet suppliers (Ma et al., 2012).

Due to its long traditional usage, *M. charantia* was subjected to several studies in humans, of which only few fulfilled the criteria of a randomized controlled trial (Leung et al., 2009). This shortcoming and the high bias associated with these studies as well as their overall low quality was emphasized in two meta-analysis of four RCTs, which revealed no significant difference in the glycemic control of bitter gourd treatment compared to placebo or untreated group, respectively (Ooi et al., 2012; Yin et al., 2014). In contrast, a systematic review of ten studies (including also non-randomized clinical trials) found a significant reduction of fasting blood glucose, post prandial glucose and HBA_1c_ in type 2 diabetic patients, however, with low quality evidence (Peter et al., 2019). All reviews conclude that further studies are warranted before bitter gourd preparations can be recommended for clinical practice.

Apart from clinical trials, the hypoglycemic effect of bitter gourd juice, fresh and dried fruit, or extracts was evaluated in repeated animal studies, reporting hypoglycemic effects for the juice as well as for water, ethanol, and acetone extracts (Singh et al., 1989; Basch et al., 2003; Krawinkel and Keding, 2006; Shetty et al., 2005). Additionally, bitter gourd was found less effective on blood glucose levels when applied as dried fruit powder (Ahmed et al., 2004), which is of interest because most clinical studies were conducted with capsules or tablets containing fruit powder rather than extracts (Efird et al., 2014). This might explain the lower outcome compared to animal studies as well as the missing standardization of the used preparations, with only two studies reporting contents of charantin (Fuangchan et al., 2011; Zänker et al., 2012). The lack of standardization was also noticed in the abovementioned meta-analysis, which demanded an increased focus on the quality control of bitter gourd preparations (Ooi et al., 2012; Peter et al., 2019).

The standardization of extracts depends on suitable marker compounds, which ideally represent the bioactive ingredient(s). In the case of *M. charantia*, early reports mentioned three active principles, namely charantin (Lotlikar and Rajarama Rao, 1966), p-insulin (Khanna et al., 1981), and vicine (Handa et al., 1990), which meanwhile are regarded controversially (Raman and Lau, 1996). Still, even recent studies and articles consider these compounds important antidiabetic agents (Desai et al., 2021; Pahlavani et al., 2019) though in the last years there has been growing evidence that the particular compound class of cucurbitanoids are responsible for the potential antidiabetic effects (Jia et al., 2017; Saeed et al., 2018; Sun et al., 2021).

In the following, potentially antidiabetic compounds isolated from *M. charantia* are summarized and discussed for their role in treating diabetes and for their use in the standardization of commercial preparations.

## Compounds of the Bitter Gourd and Diabetes-Related Activities

### Compounds With Unspecified Hypoglycemic Effects

**FIGURE 1:**
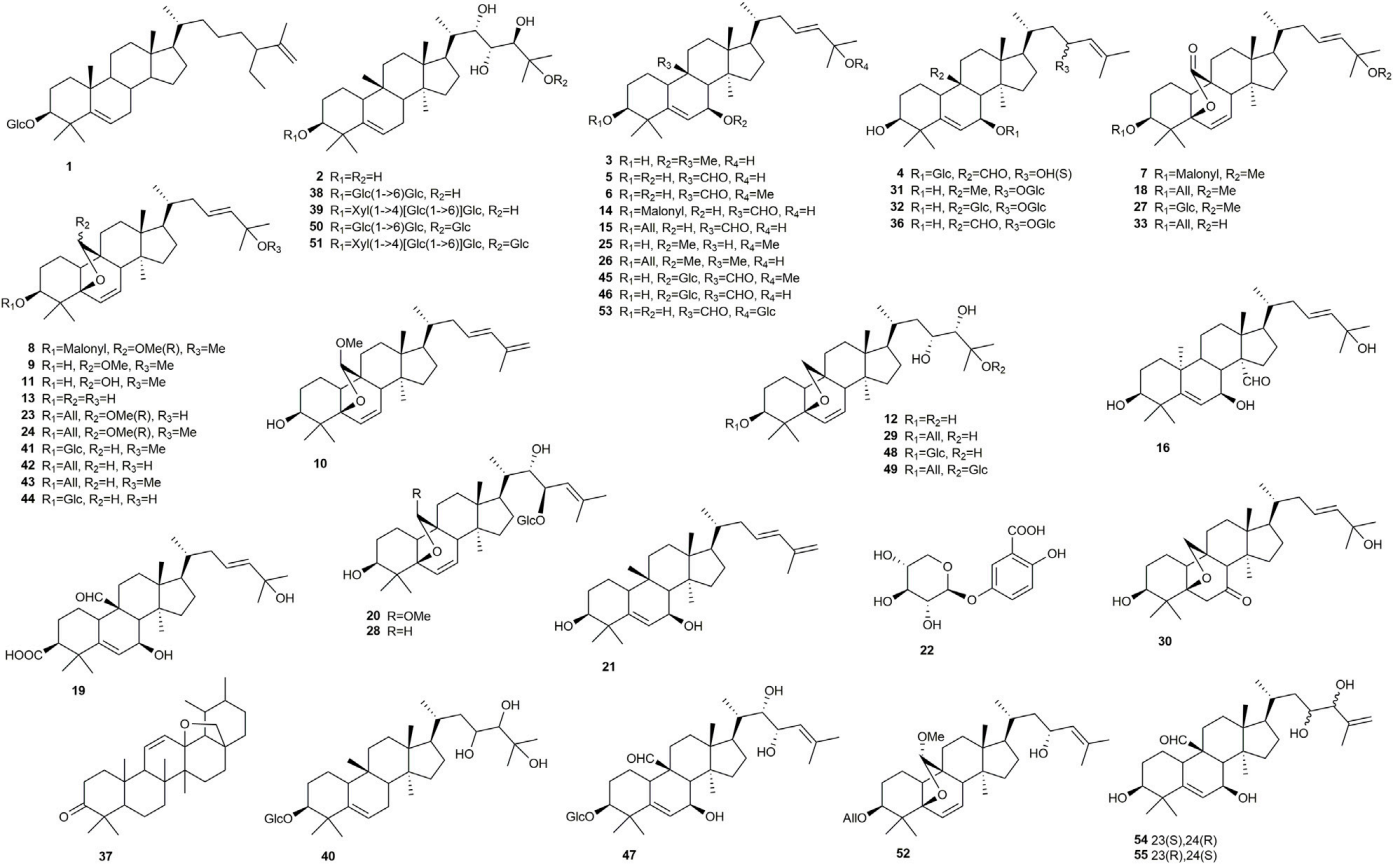
Chemical structures of compounds isolated from *Momordica charantia* with activity on targets relevant for diabetes. All means β-D-allopyranoside, Glc means β-D-glucopyranoside.

Like many other plant species of the Cucurbitaceae family, the bitter gourd contains various cucurbitane-type triterpenoids (Figure 1), with about 200 reported structures so far (Sun et al., 2021). These compounds are generally referred to as momordicosides, but also other compound names can be found, such as karavilosides, kuguacins, or goyaglycosides.

Nevertheless, the first report of a possible antidiabetic principle mentioned a mixture of the two phytosterol glycosides β-sitosterol 3-O-β-D-glucoside and 5,22-stigmasterol 3-O-β-D-glucoside, which was named charantin (Lotlikar and Rajarama Rao, 1966). Charantin elicited a hypoglycemic response in rabbits, however, at doses equivalent to 180–315 g of fruit. The same accounts for the pyrimidine nucleoside vicine, which was isolated from the seeds (Dutta et al., 1981; Barron et al., 1982). Here, administration of a dose equivalent to 16 g of seeds per kg body weight caused hypoglycemia in rats (Handa et al., 1990).

Khanna et al. (1981) described p-insulin, an 11 kDa polypeptide similar to bovine insulin, as possible antidiabetic principle. p-Insulin showed blood glucose lowering effects in patients with juvenile and maturity onset diabetes after subcutaneous administration. Another large molecule with reported antidiabetic activity is *M. charantia* polysaccharide IIa (MCPIIa, **35**) (Zhang et al., 2018). The 13 kDa polysaccharide, which is mainly composed of arabinofuranose, glucuronic acid, and xylopyranosyl residues, lowered blood glucose at doses from 100 mg/kg in STZ-induced diabetic mice (Table 1).

**TABLE 1:** Summary of reported *in vitro* and *in vivo* effects. All concentrations are given in µM, except for the measurements of blood glucose, where the administered dose is given in mg/kg body weight. Amount of stimulation or inhibition, respectively, is given in parenthesis.

	Compound Name	Blood Glucose	Glucose Uptake				Glucose Absorption		Glucose Production		Insulin Secretion	Insulin Resistance
			C2C12	FL83B	3T3-L1	AMPK	α-Amylase	α-Glucosidase	H4IIE	PEPCK	MIN6 β	PTPN2
1	25ξ-isopropenylchole-5-ene 3-O-β-d-glucopyranoside	—	—	—	—	—	870 (79%)	1,330 (71%)	—	—	—	—
2	3β,22(S),23(R),24(R),25-pentahydroxy-cucurbita-5-ene	—	—	—	1 (75%)	1	—	—	—	—	—	—
3	3β,25-dihydroxy-7β-methoxycucurbita-5,23(E)-diene	—	—	10.6 (20%)	—	21.2	—	—	—	—	—	—
4	3β,7β,23(S)-trihydroxycucurbita-5,24-dien-19-al 7-β-d-glucopyranoside	—	—	—	—	—	—	—	—	—	—	20 (72%)
5	3β,7β,25-trihydroxycucurbita-5,23(E)-dien-19-al	200 (p.o.)	—	10.6 (20%)	—	21.2	870 (69%)	1,330 (65%)	100 (55%)	—	—	—
6	3β,7β-dihydroxy-25-methoxycucurbita-5,23(E)-dien-19-al	—	—	—	—	—	—	—	100 (45%)	100 (25%)	—	—
7	3β-malonyl-25-O-methylkaravilagenin D	—	10 (26%)	—	—	—	—	—	—	—	—	—
8	5β,19-epoxy-19,25-dimethoxy-3β-malonylcucurbita-6,23-diene	—	5 (23%)	—	—	—	—	—	—	—	—	—
9	5β,19-epoxy-19,25-dimethoxycucurbita-6,23-diene-3β-ol	—	—	20.0 (50%)	—	20.0	—	—	—	—	—	—
10	5β,19-epoxy-19-methoxycucurbita-6,23(E),25-triene-3β-ol	25 (p.o.)	—	—	—	—	—	—	—	—	—	—
11	5β,19-epoxy-25-methoxycucurbita-6,23-diene-3β,19-diol	—	—	20.6 (110%)	—	20.6	—	—	—	—	—	—
12	5β,19-epoxy-3β,23(R),24(S),25-tetrahydroxy-cucurbita-6-ene	—	—	—	0.1 (80%)	0.1	—	—	—	—	—	—
13	5β,19-epoxy-3β,25-diyhdroxycucurbita-6,23(E)-diene	200 (p.o.)	—	21.9 (55%)	—	21.9	—	—	—	—	—	—
14	7β,25-dihydroxy-3β-malonylcucurbita-5,23-dien-19-al	—	10 (27%)	—	—	—	—	—	—	—	—	—
15	7β,25-dihydroxycucurbita-5,23(E)-dien-19-al 3-O-β-allopyranoside	—	10 (120%)	—	—	—	—	50 (18.63%)	—	—	—	—
16	charantal	—	—	—	—	—	870 (70%)	1,330 (60%)	—	—	—	—
17	charantin?	—	—	—	—	—	28 (50%)	—	—	—	—	—
18	charantoside C	—	—	—	—	—	—	50 (13.61%)	—	—	—	—
19	charantoside XI	—	—	—	—	—	870 (62%)	1,330 (56%)	—	—	—	—
20	charantoside XV	—	—	—	—	—	870 (68–77%)	1,330 (23.9%)	—	—	—	—
21	cucurbita-5.23(E),25-triene-3β,7β-diol	—	—	11.4 (30%)	—	22.8	—	—	—	—	—	—
22	gentisic acid 5-O-β-D-xyloside	—	—	—	—	—	870 (60.7%)	1,330 (56.4%)	—	—	—	—
23	goyaglycoside b	—	—	—	—	—	—	50 (10.88%)	—	—	—	—
24	goyaglycoside d	—	—	—	—	—	—	—	—	—	—	20 (84%)
25	karavilagenin A	—	—	—	—	—	—	—	—	—	—	20 (93%)
26	karaviloside III	—	—	—	—	—	—	50 (15.85%)	—	—	—	—
27	karaviloside VI (charantoside X)	—	—	—	—	—	870 (68–77%)	1,330 (40.3%)	—	—	—	—
28	karaviloside VIII	—	—	—	—	—	870 (68–77%)	1,330 (56.6%)	—	—	—	—
29	karaviloside XI	—	—	—	0.1 (80%)	0.1	870 (66.4%)	1,330 (49.4%)	—	—	—	—
30	kuguacin II	—	—	—	—	—	—	—	100 (50%)	—	—	—
31	kuguaglycoside A	—	—	—	—	—	—	—	100 (45%)	100 (25%)	—	—
32	kuguaglycoside G	—	—	—	—	—	—	—	—	—	12.8 (8.1-fold)	—
33	kuguaglycoside I	—	10 (22%)	—	—	—	—	—	—	—	—	—
34	mcIRBP	0.02 (i.p.)	—	—	3.92 (50%)	—	—	—	—	—	—	—
35	MCPIIa	100 (p.o.)	—	—	—	—	—	—	—	—	—	—
36	momordicine II	—	—	—	—	—	—	—	—	—	15.5 (7.3-fold)	—
37	momordicinin	—	—	—	—	—	36 (50%)	—	—	—	—	—
38	momordicoside A	—	—	—	—	—	870 (68–77%)	50 (21.71%) 1,330 (33.5%)	—	—	—	—
39	momordicoside B	—	—	—	10 (30%)	—	—	—	—	—	—	—
40	momordicoside C	—	—	—	—	—	—	50 (12.98%)	—	—	—	—
41	momordicoside F1	—	—	—	—	—	870 (63.5%)	50 (11.51%) 1,330 (35.9%)	—	—	—	20 (73%)
42	momordicoside F2	—	—	—	—	—	870 (63.7%)	50 (12.50%) 1,330 (42.4%)	—	—	—	—
43	momordicoside G	—	—	—	—	—	870 (70.5%)	50 (15.14%) 1,330 (48.5%)	—	—	—	—
44	momordicoside I	—	—	—	—	—	870 (64.2%)	50 (10.11%) 1,330 (35.1%)	—	—	—	—
45	momordicoside K	—	10 (40%)	—	—	—	870 (69.0%)	1,330 (47.5%)	—	—	—	20 (84%)
46	momordicoside L	—	—	—	—	—	870 (68–77%)	1,330 (23.7%)	—	—	—	20 (72%)
47	momordicoside M	—	—	—	—	—	—	50 (18.63%)	—	—	—	—
48	momordicoside Q	—	—	—	10 (35%)	—	—	—	—	—	—	—
49	momordicoside R	—	—	—	10 (35%)	—	—	—	—	—	—	—
50	momordicoside S	100 (i.p.)	—	—	0.01 (50%)	0.1	—	—	—	—	—	—
51	momordicoside T	10 (i.p.)	—	—	10 (50%)	—	—	—	—	—	—	—
52	momordicoside U	—	—	—	—	—	—	—	—	—	—	20 (72%)
53	momordicoside X	—	—	—	—	—	—	—	—	—	15.8 (2.7-fold)	—
54	yeojooside G	—	—	—	—	—	—	—	—	—	—	20 (93%)
55	yeojooside H	—	—	—	—	—	—	—	—	—	—	20 (87%)

It was 40 years after the first report of charantin, when Harinantenaina et al. (2006) reported blood glucose-lowering effects for two cucurbitane-type triterpenoids (**5**, **13**) in alloxan-induced diabetic mice. Both compounds were obtained from the ether-soluble fraction of a methanol extract and were significantly active upon oral administration of 200 mg/kg. In the same study β-sitosterol and 5,22-stigmasterol, the two aglycones of charantin, exhibited no effect. Jiang et al. (2016) demonstrated blood glucose and triglyceride lowering effects for another cucurbitane-type triterpenoid (**10**), which was administered at a dose of 25 mg/kg by oral gavage over a period of 4 weeks.

### Compounds Enhancing Glucose Uptake

Tan et al. (2008) reported eight cucurbitane-type triterpenoids (**2**, **12**, **29**, **39**, **48**–**51**) with enhanced GLUT4-translocation in L6 myotubes and 3T3-L1 adipocyte cells. Karaviloside XI (**29**) and momordicoside S (**50**) as well as their agylcones (**2**, **12**) were the most active components, with the observed effects starting at a concentration of 0.1 nM and reaching their maximum between 10 and 100 nM. The study furthermore revealed that GLUT4-translocation was mediated *via* AMPK phosphorylation and not *via* the PI3K/Akt pathway. Momordicoside S (**50**) and momordicoside T (**51**) were further studied for their hypoglycemic effects *in vivo*. At doses of 100 mg/kg (**50**) and 10 mg/kg **51**) both compounds showed a significantly higher glucose clearance than the traditional AMPK agonist AICAR. In addition, momordicoside T (**51**) lowered blood glucose levels in insulin-resistant high-fat-fed mice.

Cheng et al. (2008) investigated the effect of extracts and fractions from the stems of *M. charantia* in hepatic FL83B cells leading to the isolation of three triterpenoids (**3**, **5**, **21**). At a dose of 5 μg/ml, all three compounds enhanced glucose uptake comparably to insulin-treated cells. The study, moreover, showed that the effect was mediated *via* AMPK and that the compounds reduced insulin resistance in cells. AMPK was also the target of a study by Chang et al. (2011), who identified three effective triterpenoids (**9**, **11**, **13**). While the activity of compounds **9** and **13** was close to that of the control troglitazone, the effect of **11** was even greater.

Hsiao et al. (2013) investigated 15 cucurbitane type triterpenoids from bitter gourd fruits in C2C12 myoblasts. At a concentration of 10 μM, two components (**15** and **45**) enhanced glucose uptake by 50% and more than 100%, respectively. The latter compound was even more effective than the positive control insulin. Han et al. (2018) investigated another four triterpenoids (**7**, **8**, **14**, **33**) in C2C12 cells. At the highest concentrations, all four compounds increased glucose uptake from 22 to 27% compared to control. The effect was even more pronounced in insulin-stimulated cells, with values of 29% (**7**, **14**), 48% (**8**), and 39% (**33**).

The most recent study was done by Lo et al. (2013), who isolated *M. charantia* insulin receptor-binding protein (mcIRBP, **34**). The polypeptide stimulated glucose uptake into 3T3-L1 cells and significantly decreased blood glucose levels upon intraperitoneal injection (Lo et al., 2014). In order to eventually increase oral bioavailability of the newly identified peptide, mcIRBP (**34**) was digested with pepsin in a follow-up study (Lo et al., 2016). Thereby, a 19 amino-acid spanning residue (mcIRBP-19) was identified as blood glucose-lowering motif with even increased stimulation of glucose uptake. In a recent RCT, the effect of an aqueous bitter gourd preparation containing 0.17% mcIRBP-19 on fasting blood glucose and HbA1c levels was investigated (Yang et al., 2022). The results were of only borderline significance, however, a subgroup not responding to antidiabetic medication was affected more significantly.

### Compounds Targeting Glucose Absorption

Nhiem et al. (2010) tested 14 cucurbitane glycosides for their inhibition of α-glucosidase. Eight of the isolated compounds (**15**, **18**, **23**, **26**, **40**, **41**, **44**) showed weak activity at a concentration of 50 μM, whereas momordicosides A (**38**) and M (**47**) exhibited moderate inhibitory effects. The latter two compounds were the most polar components, each possessing two glycoside moieties as well as four hydroxyl groups in position C-22, C-23, C-24, and C25. Kulkarni et al. (2021) investigated the effect of charantin (**17**) and momordicinin (**37**) on the enzyme α-amylase with IC_50_ values of 28 and 36 μM, respectively. However, with regard to charantin (**17**) the results have to be regarded with caution as the structure given in the publication shows a different molecule and no details on the identification of the compound are given.

α-Glucosidase and α-amylase were also the target of three recent studies done by the same group (Shivanadoudra et al., 2019a and, 2019b; Perera et al., 2021). Thereby, a total of 15 triterpenoids (**1**, **5**, **16**, **19**, **20**, **27**–**29**, **38**, **41**–**46**) and one phenolic compound (**22**) were tested at (relatively high) concentrations of 1.33 and 0.87 mM, respectively. The inhibitory effects against α-glucosidase were ranging from 24 to 71% while the activity against α-amylase was determined from 61 to 79%. In both assay, compound **1** showed the strongest inhibition.

### Compounds Targeting Glucose Production

Chen et al. (2015) investigated 21 cucurbitane-type triterpenoids for their effect on glucose production in hepatic H4IIE cells. At a concentration of 100 μM, four triterpenoids (**5**, **6**, **30**, **31**) were inhibiting gluconeogenesis by approximately 50%. At the same concentration, **6** and **31** reduced PEPCK activity, however, by only about 25% compared to untreated control.

### Compounds Stimulating Insulin Secretion

Ma et al. (2010) investigated momordicoside X (**53**) for its effect on insulin secretion in MIN6 β-cells and found the compound to be active at a concentration of 15.8 µM. Of note, the compound was named momordicoside U in the original publication though this compound name was used before by Nhiem et al. (2010) for another structure (**52**). This fact was later clarified in the respective Erratum (Planta Medica, 2012). In a follow-up study, another five cucurbitane-type triterpenoids, two aglycones and three glycosides, were evaluated in the same assay (Keller et al., 2011). The monodesmoside momordicine II (**36**) and the bisdesmoside kuguaglycoside G (**32**) were both effective at concentrations of 10 and 25 μM, respectively, while the third glycoside and the two tested aglycones did not enhance insulin secretion. Interestingly, one of the inactive components was the aglycone of momordicoside X (**53**).

### Compounds Targeting Insulin Resistance

In a recent study, Lee et al. (2021) tested 22 cucurbitanoids for their inhibition of PTPN2, an enzyme associated with insulin resistance. At a concentration of 20 μM, nine compounds (**4**, **24**, **25**, **41**, **45**, **46**, **52**, **54**, **55**) showed inhibitory activity ranging from 72 to 93%. Additionally, all of the isolates were tested for their inhibition of α-glucosidase, however, with no effect compared to acarbose.

## Quality Control and Standardization of Extracts

Several methods for the quality control of *M. charantia* fruits and preparations have been reported. Wang et al. (2008) quantified the amount of five cucurbitane-type triterpenoids (**5**, **38**, **42**, **45**, **46**) in three different populations by HPLC-ELSD, while Ma et al. (2012) determined compounds **5**, **38**, **45**, **46**, and momordicine I in ten commercial products by HPLC-MS/MS. Other methods were dealing with the quantitation of charantin by HPTLC (Ahamad et al., 2015) and HPLC-UV (Desai et al., 2021) or with the determination of total fatty acid, saponin, and/or phenolic content (Habicht et al., 2011; Tan et al., 2014). Mishra et al. (2022) presented an NMR-based metabolomic approach for the differentiation of pericarp and seeds from the fruit skin by the abundance of phenolic compounds, charantin, and specific amino acids. However, pericarp and seed profiles were not clearly separated. Moreover, mature fruits were used in this study and no attention was paid to cucurbitane glycosides.

As explained above, the evidence for charantin as active principle is very low, wherefore its use for the standardization of extracts must be questioned. Apart from that, there is not one or a few single components that explain the bioactivity, but a plethora of cucurbitanoids acting on different targets. Thus, the HPLC methods reported by Wang et al. (2008) and Ma et al. (2012) seem most appropriate for the quality control of bitter gourd fruits and preparations. Each of the two methods determines one bidesmoside, three monodesmosides, and one aglycone, a ratio that quite well represents the glycosylation pattern in bitter gourd fruits. At the same time, the quantitation of five components is not too complex but indeed seems necessary with regard to the varying amounts in both, plant populations and commercial products. Especially, the comparison of ten different market products revealed distinct differences in the estimated daily intake of triterpenoids, with values ranging from 17 to 6,927 µg/day (Ma et al., 2012). The calculation of the estimated daily intake might also be a valuable tool for the standardization of extracts. Here, a daily intake of e.g., 3 mg cucurbitane-type triterpenoids may be a reasonable amount, which was both the mean and median of the investigated commercial preparations.

## Toxicological Properties and Safety Considerations

Early animal studies on the reproductive effects of *M. charantia* found a reduced fertility rate of female mice upon administration of fresh leave juice (Stepka et al., 1974). Furthermore, reduced spermatogenesis was reported in male rats and dogs after feeding of seed and fruit extracts at doses of 250 and 97–167 mg/kg, respectively (Dixit et al., 1978; Naseem et al., 1998). Additionally, uterine bleeding in pregnant rats and rabbits was induced with 6 ml/kg fresh fruit juice (Raman et Lau, 1993). Hsu et al. (2011) investigated a potential estrogenic effect for the reduced fertility rate by testing six isolated triterpenoids in an estradiol transactivation assay. Four of the tested compounds showed partial agonistic and/or antagonistic activity *via* α- and β-estrogen receptors. A more recent study investigated the effect of *M. charantia* fruit and seed extracts on developmental toxicity in zebra fish embryos (Khan et al., 2019). Thereby, a crude seed extract was lethal with LD_50_ values of 50 μg/ml, whereas the fruit extract did not result in any lethality up to 200 μg/ml. However, at a concentration of 50 μg/ml, cardiac toxicity for the embryo was observed, which was also much more pronounced for the seed extract (5 μg/ml).

Studies on the toxicity in humans did not results in serious adverse effects. However, two reports observed hypoglycemic coma of two children aged three and four after drinking a tee of the leaves and vines before food consumption (Raman and Lau, 1996). With regard to toxic constituents, a mildly toxic lectine was reported in the seeds and outer rind or the fruits (Marles and Farnsworth, 1997). Moreover, the alkaloid vicine in the seeds causes favism in people suffering from glucose-6-phosphate dehydrogenase deficiency (Mager et al., 1965). Therefore, decoctions of the seeds and other parts of the plant should be regarded with caution. The most common traditional mode of application is the juice of the unripe fruits, for which no serious adverse events have been reported. Moreover, the fruits are regularly consumed in Asian cuisine. Nevertheless, some studies reported uterine bleeding in pregnant animals and the estrogenic activity of four bitter gourd triterpenoids was confirmed. Therefore, like most other drugs, also bitter gourd preparations should be omitted during pregnancy.

## Conclusions and Outlook

The long traditional use and the mainly positive results from *in vivo* studies suggest beneficial effects for bitter gourd preparations in the treatment of diabetes. Continuous studies on cucurbitane-type triterpenoids point towards this compound class as active principle, with several of the investigated compounds exhibiting pronounced effects in glucose uptake assays at nanomolar (**2**, **12**, **29**, **39**) to low micromolar (**15**, **45**) concentrations. Furthermore, some of the tested compounds (**3**, **5**, **9**, **11**, **13**, **21**) showed insulin-sensitizing effects comparable to thiazolidinediones as well as enhanced insulin secretion (**32**, **36, 53**). Moreover, a recent study found several compounds to target insulin resistance *via* protein tyrosine phosphatase inhibitory activity. Apart from their abundance in bitter gourd fruits, triterpenoids (and especially their glycosylated forms) tend to dissolve in many solvents commonly used for the preparation of extracts, e.g., acetone, ethanol, and water, or mixtures thereof. This may explain the activity of different types of extracts and furthermore corroborates the cucurbitane-type triterpenoids most significantly contributing to the antidiabetic effect. Still, most clinical trials did not reflect the antidiabetic effect, which may be due to the use of fruit powder rather than extracts in these studies and the varying cucurbitanoid contents of commercial products. In addition, missing standardization of the used preparations or standardization with an inadequate maker might be an explanation for the inconsistent pharmacological results. Thus, future studies should apply formulations (preferably made of extracts) with a defined amount of daily intake. Considering the safety of bitter gourd preparations, the use of fruits free from seeds might reduce the risk of adverse events.
